# Pediatric tuberculosis outcomes and factors associated with unfavorable treatment outcomes in Botswana, 2008–2019: a retrospective analysis

**DOI:** 10.1186/s12889-022-14477-y

**Published:** 2022-11-04

**Authors:** Keatlaretse Siamisang, Goabaone Rankgoane-Pono, Tumisang Malebo Madisa, Tantamika Kabamba Mudiayi, John Thato Tlhakanelo, Paul Mubiri, Khutsafalo Kadimo, Francis Msume Banda, Vincent Setlhare

**Affiliations:** 1 Department of Family Medicine and Public Health, University of Botswana, Gaborone, Botswana; 2 Department of Health Services Management, Ministry of Health, Gaborone, Botswana; 3 Maryland Global Initiative Corporation (MGIC), University of Maryland, Baltimore (UMB), Gaborone, Botswana; 4 Department of Library Services, University of Botswana, Gaborone, Botswana; 5 Department of Pediatrics, University of Botswana, Gaborone, Botswana; 6 P O Box 40, Letlhakeng, Botswana

**Keywords:** Pediatric, Tuberculosis, Outcomes, Associated factors, Botswana

## Abstract

**Introduction:**

Globally, the amount of research on the outcomes of pediatric tuberculosis (TB) is disproportionately less than that of adult TB. The diagnosis of paediatric TB is also problematic in developing countries. The aim of this study was to describe the outcomes of pediatric TB in Botswana and to identify the factors associated with unfavorable outcomes.

**Methods:**

This was a retrospective analysis of pediatric TB outcomes in Botswana, over a 12-year period from January 2008 to December 2019. Treatment success (treatment completion or cured) was considered a favorable outcome, while death, loss to follow-up and treatment failure were considered unfavorable outcomes. Program data from drug-sensitive TB (DS-TB) cases under the age of 15 years were included. Sampling was exhaustive. Binary logistic regression was used to determine the factors associated with unfavorable outcomes during TB treatment. A p value of < 0.05 was considered a statistically significant association between the predictor variables and unfavorable outcomes.

**Results:**

The data of 6,004 paediatric TB cases were extracted from the Botswana National TB Program (BNTP) electronic registry and analyzed. Of these data, 2,948 (49.4%) were of female patients. Of the extracted data, 1,366 (22.8%) were of HIV positive patients and 2,966 (49.4%) were of HIV negative patients. The rest of the data were of patients with unknown HIV status. Pulmonary TB accounted for 4,701 (78.3%) of the cases. Overall, 5,591 (93.1%) of the paediatric TB patient data showed treatment success, 179 (3.0%) were lost to follow-up, 203 (3.4%) records were of patients who died, and 31 (0.5%) were of patients who experienced treatment failure. The factors associated with unfavorable outcomes were positive HIV status (AOR 2.71, 95% CI: 2.09–3.52), unknown HIV status (AOR 2.07, 95% CI: 1.60–2.69) and retreatment category (AOR 1.92, 95% CI: 1.30–2.85). Compared with the 0–4 years age category, the 5–9 years (AOR 0.62, 95% CI: 0.47–0.82) and 10–14 years (AOR 0.76, 95% CI: 0.60–0.98) age categories were less likely to experience the unfavorable outcomes.

**Conclusion:**

This study shows a high treatment success rate among paediatric TB cases in Botswana. The government under the National TB Program should maintain and consolidate the gains from this program. Public health interventions should particularly target children with a positive or unknown HIV status, those under 5 years, and those who have been previously treated for TB.

**Supplementary Information:**

The online version contains supplementary material available at 10.1186/s12889-022-14477-y.

## Introduction

The World Health Organization (WHO) declared tuberculosis (TB) a global emergency in 1993. Multiple papers on the burden, outcomes, and control of TB have since been published. However, the focus has mostly been on adult disease [1]. Globally, there appears to be less research on pediatric TB and this has resulted in limited data on pediatric TB burden and outcomes. Furthermore, pediatric TB can be difficult to diagnose, especially in the context of HIV infection [2, 3]. However, it remains a public health concern, especially in developing countries. About 10% of all TB patients are children under the age of 15 years [4]. In developing countries, 20% of TB notifications are in this age group [3, 5]. Pediatric TB usually indicates a recent transmission and a source of infection in the community [4]. Young children usually become infected following exposure to an infected adult in their household. However, in endemic areas, significant transmission occurs outside of the household [5]. Pediatric TB is therefore an important indicator of the performance of TB control programs [6].

Botswana is a Southern African country with a high burden of both HIV and TB [7–9]. This possibly affirms that HIV infection is the most important risk factor for TB infection and disease [10]. According to the United Nations Joint Programme on HIV/AIDS (UNAIDS) 2020 estimate, the country had an HIV prevalence of 19.9% among 19 to 45-year-olds and an HIV incidence of 4.4% [11]. The HIV/TB coinfection rate in adults is estimated at 60%, which is consistent with claims that HIV and TB are bidirectional and synergic [12]. Nonetheless, there is limited data on pediatric TB in Botswana. Data from an electronic TB registry in 1998–2002 revealed that 67% of patients had favorable outcomes (cured or completed treatment) while 10.5% died [13]. Predictors of death in this study were age below 5 years, male gender, having an HIV positive mother or mother of unknown HIV status, alcohol use by the mother, and reported adverse TB drug effects [13]. This study was limited to only 2 districts. There is a need for an up-to-date nationally representative study of pediatric TB outcomes and their predictors in Botswana. Paediatric TB outcomes have been described in other Sub-Saharan countries. In a Ghanaian study in 2013, the treatment success rate was 90% and the mortality rate was 8.4%. HIV positivity and smear positive TB were predictors of mortality [14]. The success rate was 83% in a 2015 Nigerian study. HIV status was a predictor of mortality [15]. Other African studies have reported comparable success rates [16, 17].

Since TB is a curable disease, it is important to know the predictors of adverse outcomes among children treated for TB so that appropriate interventions may be implemented. This may help save the lives of many children. Hence, this study focused on describing the outcomes of pediatric TB in Botswana and identifying the factors associated with unfavorable outcomes in children under the age of 15 years.

## Methods

### Study Design and population

This was a retrospective analysis of paediatric TB data for the period from 01 to 2008 to 31 December 2019. The data was captured from the Botswana National TB Program (BNTP) electronic registry. The BNTP database contains paediatric TB data from all health facilities in Botswana. The data of interest was that of patients under the age of 15 years who were started on a standard TB treatment regimen via directly observed therapy. The diagnostic algorithm of TB in BNTP includes both sputum microscopy and gene Xpert. However, the challenges to capturing Xpert data into the national electronic records system mean this data is not available for most of the study period. The TB diagnosis can also be made based on clinical and radiological findings. This data is not captured on the electronic medical records system.

### Study setting

The study included all paediatric drug-sensitive TB (DS-TB) notifications in Botswana during the study period. All health districts were included in the study. In Botswana, the management of TB is fully integrated into the Primary Health Care (PHC) through district hospitals, clinics, and health posts. The data is documented in the facility TB registers. Each health district has a TB coordinator who inputs all TB surveillance data into the electronic medical records system called Open Medical Records System (Open MRS).

## Variables

Data were extracted from the BNTP electronic registry. The variables that were extracted included patient age, sex, HIV status, TB classification (site of infection), disease category (new/retreatment), microscopy results, and TB treatment outcomes. The TB treatment outcomes are determined and entered by the TB coordinators at the district level. The coordinators use data from facility TB registers and apply the World Health Organization (WHO) definitions of treatment outcomes for DS-TB [18]. Some of the variables in the facility TB registers are not captured on the national electronic medical records system. This paper uses the captured outcomes. The following definitions are used by the TB coordinators to determine the treatment outcomes.

- Cured: A pulmonary TB patient with bacteriologically confirmed TB at the beginning of treatment who was smear or culture-negative in the last month of treatment and on at least 1 previous occasion.
- Treatment Completed: A TB patient who completed treatment without evidence of failure BUT with no record to show that sputum smear or culture results in the last month of treatment and on at least one previous occasion were negative either because tests were not done or results are not available.
- Treatment failed: A TB patient whose sputum smear or culture were positive at month 5 of treatment or later.
- Died: A TB patient who dies for any reason before starting or during treatment.
- Lost to follow-up: A TB patient who did not start treatment or whose treatment was interrupted for two consecutive months or more.
- Not evaluated: A TB patient for whom no treatment outcome is assigned.
- Treatment Success: The sum of cured and treatment completed.

In this study, treatment success was considered a favorable outcome, while death, loss to follow-up and treatment failure were considered unfavorable outcomes.

### Data Analysis

Statistical analyses were performed using STATA software version 17 (Stata Corp, College Station, TX, USA). The analysis was limited to the variables that are routinely captured in the electronic records system. Frequencies, medians, and interquartile ranges were used to summarize the data. Crude and adjusted odds ratios (OR) with 95% confidence intervals (CI) for unfavorable outcomes (death, loss to follow-up, treatment failure) were calculated using a logistic regression model stratified by age. Factors significant at 10% in the univariable analysis as well as factors with plausible association with each outcome were included in the multivariable model. Since the effect of the predictor variables on the outcome variable could have changed over the 12-year period, the year of notification was controlled for in the multivariable model. Collinearity was assessed using variance inflation factors. The models were assessed for goodness of fit using the Log-likelihood ratio test, Akaike Information Criteria (AIC), and Bayesian Information Criteria (BIC). A parsimonious model was reported. Kaplan Meier curves were used to display cumulative mortality for the 3 age categories (0–4 years, 5–9 years, and 10–14 years). The follow-up time was the number of days treated and the outcome (failure) was mortality. TB cases that were lost to follow-up were censored. The log rank test was used to compare survival between the different age categories.

## Results

Data from 6,004 paediatric TB patients under the age of 15 years were included in the analysis. Of these, 2,948 (49.1%) were female patients. The data comprised 3,054 records of patients aged 0–4 years, 1,371 aged 5–9 years, and 1,579 aged 10–14 years. The data showed characteristics of the cases as displayed in Table 1. In the data, there were 1,366 (22.8%) records of HIV-positive patients, and 2,966 (49.4%) records were of HIV-negative cases. The rest (27.9%) had an unknown HIV status. HIV status was positive in 17.0%, 24.7%, and 32.2% of 0–4 years, 5–9 years, and 10–14 years age groups, respectively. In terms of disease classification, 4,701 (78.3%) of the records were of pulmonary TB patients. The classification was pulmonary in 79.7%, 75.0%, and 78.5% of 0–4 years, 5–9 years, and 10–14 years age groups, respectively. A total of 5,523 (92.0%) TB records were of new TB cases. This category accounted for 93.1%, 92.5%, and 89.4% of 0–4 years, 5–9 years, and 10–14 years age groups, respectively. In the total group, 5,591 (93.1%) records showed treatment success, 179 (3.0%) showed loss to follow-up, 203 (3.4%) were of patients who died and 31 (0.5%) were of patients who failed treatment. In the 0–4 years age group, 2,823 (92.4%) had treatment success, 93 (3.1%) were lost to follow-up, 129 (4.2%) died and 9 (0.3%) failed treatment. In the 5–9 years age group, 1,298 (94.7%) achieved treatment success, 36 (2.6%) were lost to follow-up, 30 (2.2%) died and 7 (0.5%) had treatment failure. Finally, in the 10–14 age group, 1,470 (93.1%) achieved treatment success, 50 (3.2%) were lost to follow-up, 44 (3.0%) died and 7 (0.5%) had treatment failure.

**Table 1:** Characteristics and outcomes of pediatric TB cases, 2008–2019 (n = 6,004)

Variable, n (%)	0–4 years (n = 3,054)	5–9 years (n = 1,371)	10–14 years (n = 1,579)	Total (n = 6,004)
**Sex**				
*Data available*	*3,054 (100)*	*1,371 (100)*	*1,579 (100)*	*6,004 (100)*
Females	1,384 (45.3)	658 (48.0)	906 (57.4)	2,948 (49.1)
Males	1,670 (54.7)	713 (52.0)	673 (42.6)	3,056 (50.9)
**HIV status**				
*Data available*	*3,054 (100)*	*1,371 (100)*	*1,579 (100)*	*6,004 (100)*
HIV negative	1,595 (52.2)	674 (49.2)	697 (44.1)	2,966 (49.4)
HIV positive	518 (17.0)	339 (24.7)	509 (32.2)	1,366 (22.8)
Status unknown	941 (30.8)	358 (26.1)	373 (23.6)	1,672 (27.9)
**Disease classification**				
*Data available*	*3,054 (100)*	*1,369 (99.8)*	*1,578 (100)*	*6,001 (100)*
Pulmonary	2,435 (79.7)	1,027 (75.0)	1,239 (78.5)	4,701 (78.3)
Extra-pulmonary	619 (20.3)	342 (25.0)	339 (21.5)	1,300 (21.7)
**Treatment category**				
*Data available*	*3,054 (100)*	*1,371 (100)*	*1,579 (100)*	*6,004 (100)*
New	2,843 (93.1)	1,268 (92.5)	1,412 (89.4)	5,523 (92.0)
Retreatment	211 (6.9)	103 (7.5)	167 (10.6)	581 (8.0)
**Sputum microscopy**				
*Data available*	*2,575 (84.3)*	*1,211(88.3)*	*1,341(84.9)*	*5,127 (85.4)*
Positive	317 (12.3)	189 (15.6)	484 (36.1)	990 (19.3)
Negative	319 (12.4)	176 (14.5)	249 (18.6)	744 (14.5)
Microscopy not done	1,939 (75.3)	846 (69.9)	608 (45.3)	3,393 (66.2)
**Treatment outcome**				
*Data available*	*3,054 (100)*	*1,371 (100)*	*1,579 (100)*	*6,004 (100)*
Treatment success	2,823 (92.4)	1,298 (94.7)	1,470 (93.1)	5,591 (93.1)
Loss to follow up (LTFU)	93 (3.1)	36 (2.6)	50 (3.2)	179 (3.0)
Died	129 (4.2)	30 (2.2)	44 (3.0)	203 (3.4)
Failed	9 (0.3)	7 (0.5)	15 (1.0)	31 (0.5)
Unfavorable outcome (LTFU, died, failed)	231 (7.6)	73 (5.3)	109 (7.2)	413 (6.9)

Table 2 shows the antiretroviral (ART) status of the HIV positive patients. Of the 1366 patients, 753 (55.1%) were on ART and 137 (10.0%) were not on ART. ART status was unknown for 476 (34.9) of the patients.

**Table 2:** Anti-retroviral (ART) status of HIV positive paediatric TB patients 2008–2019 (n = 1,366)

Anti-retroviral status	0–4 years	5–9 years	10–14 years	Total
*Data available*	*518 (100)*	*339 (100)*	*509 (100)*	*1366 (100)*
On ART	290 (56.0)	184 (54.3)	279 (54.8)	753 (55.1)
Not on ART	47 (9.1)	35 (10.3)	55 (10.8)	137 (10.0)
ART status unknown	181 (34.9)	120 (35.4)	175 (34.4)	476 (34.9)

Between 2008 and 2012, completion and cure rates could not be separated in the data from the electronic records system. We therefore report treatment success rates in Table 1. Table 3 shows the treatment outcomes from 2013 to 2019. In this period, completion and cure rates are reported separately. Out of the 2,644 cases, 2,280 (86.2%) completed treatment, 216 (8.2%) were cured, 49 (1.9%) were lost to follow-up, 90 (3.4%) died and 9 (0.3%) failed treatment. In the 0–4 years old age group, 1,282 (89.1%) completed treatment, 61 (4.2%) were cured, 33 (2.3%) were lost to follow-up, 60 (4.2%) died and 3 (0.2%) failed treatment. In the 5–9 years age group, 464 (88.4%) completed treatment, 44 (8.0%) were cured, 6 (1.1%) were lost to follow-up, 11 (2.1%) died and 2 (0.4%) failed treatment. In the 10–14 year age group, 534 (78.5%) completed treatment, 113 (16.6%) were cured, 10 (1.5%) were lost to follow-up, 19 (2.8%) died and 4 (0.6%) failed treatment.

**Table 3:** Pediatric tuberculosis outcomes according to age category, 2013–2019 (n = 2,644)

Outcome	0–4 years (n = 1,439)	5–9 years (n = 525)	10–14 years (n = 680)	Total (n = 2,644)
Completed	1,282 (89.1)	464 (88.4)	534 (78.5)	2,280 (86.2)
Cured	61 (4.2)	44 (8.0)	113 (16.6)	216 (8.2)
Loss to follow up	33 (2.3)	6 (1.1)	10 (1.5)	49 (1.9)
Died	60 (4.2)	11 (2.1)	19 (2.8)	90 (3.4)
Failed	3 (0.2)	2 (0.4)	4 (0.6)	9 (0.3)
Total	1,439 (100)	525 (100)	680 (100)	2,644 (100)

Table 4 shows the factors associated with unfavorable outcomes in the multivariable model for pediatric patients of all ages from 2008 to 2019. TB patients who died, failed treatment, or were lost to follow-up were considered to have unfavorable outcomes in this analysis. Compared to the 0–4 -year-old age group, cases aged 5–9 years were less likely to have unfavorable outcomes (AOR 0.62, 95% CI: 0.47–0.82). Cases aged 10–14 years were also less likely to have unfavorable outcomes (AOR 0.76, 95% CI: 0.60–0.90). Compared to HIV-negative cases, HIV positive cases (AOR 2.71, 95% CI: 2.09–3.52) and cases with an unknown HIV status (AOR 2.07, 95% CI: 1.60–2.69) were more likely to have an unfavorable outcome. Compared to new cases, retreatment cases (AOR 1.92, 95% CI: 1.30–2.85) were more likely to have unfavorable outcomes.

**Table 4:** Crude and adjusted odds ratios for unfavorable tuberculosis treatment outcomes in all pediatric age groups, 2008–2019 (n = 6,004)

Variable	Crude odds ratio	p value	Adjusted odds ratio	p value
**Male sex**	1.11 (0.91–1.35)	0.318	1.09 (0.89–1.34)	0.412
**Age category**				
0–4 years	Reference			
5–9 years	0.69 (0.52–0.90)	0.007	0.62 (0.47–0.82)	0.001
10–14 years	0.91 (0.72–1.15)	0.414	0.76 (0.60–0.98)	0.033
**HIV status**				
HIV negative	Reference			
HIV positive	2.70 (2.10–3.47)	< 0.001	2.71 (2.09–3.52)	< 0.001
Status unknown	2.22 (1.74–2.85)	< 0.001	2.07 (1.60–2.69)	< 0.001
**Disease classification**				
Pulmonary	Reference			
Extra-pulmonary	1.09 (0.86–1.38)	0.476	1.14 (0.90–1.46)	0277
**Treatment Category**				
New	Reference			
Retreatment	1.57 (1.14–2.15)	0.005	1.92 (1.30–2.85)	0.001

Table 5 shows the factors associated with unfavorable outcomes in the different age groups. Among the 0–4 year olds, the significant predictors of unfavorable outcomes were positive HIV status (AOR 3.54, 95% CI: 2.49–5.02) and unknown HIV status (AOR 2.35, 95% CI: 1.69–3.28). Among the 5–9 year olds, the significant predictor of unfavorable outcomes was positive HIV status (AOR 2.04, 95% CI: 1.14–3.63). Among the 10–14 year olds, the predictors of mortality were positive HIV status (AOR 2.08, 95% CI: 1.25–3.44), unknown HIV status (AOR 2.20, 95% CI: 1.29–3.79) and retreatment category (AOR 2.38, 95% 1.33–4.29).

**Table 5:** Factors associated with unfavorable tuberculosis treatment outcomes in different pediatric age categories 2008–2019 (n = 6,004)

	0–4 year old		5–9 years old		10–14 year old	
	**Adjusted odds ratio**	**p value**	**Adjusted odds ratio**	**p value**	**Adjusted odds ratio**	**p value**
**Male sex**	1.01 (0.77–1.33)	0.917	0.97 (0.60–1.57)	0.91	1.35 (0.91–2.01)	0.139
**HIV status**						
HIV negative	Reference		Reference		Reference	
HIV positive	3.54 (2.49–5.02)	< 0.001	2.04 (1.14–3.63)	0.016	2.08 (1.25–3.44)	0.004
Status unknown	2.35 (1.69–3.28)	< 0.001	1.74 (0.96–3.15)	0.070	2.20 (1.29–3.79)	0.004
**Disease classification**						
Pulmonary	Reference		Reference		Reference	
Extra-pulmonary	1.23(0.73–2.08)	0.428	0.79 (0.44–1.41)	0.419	1.25 (0.78–1.99)	0.352
**Category**						
New	Reference		Reference		Reference	
Retreatment	1.27 (0.92–1.76)	0.149	1.72 (0.77–3.83)	0.188	2.38 (1.33–4.29)	0.004

**Fig. 1:**
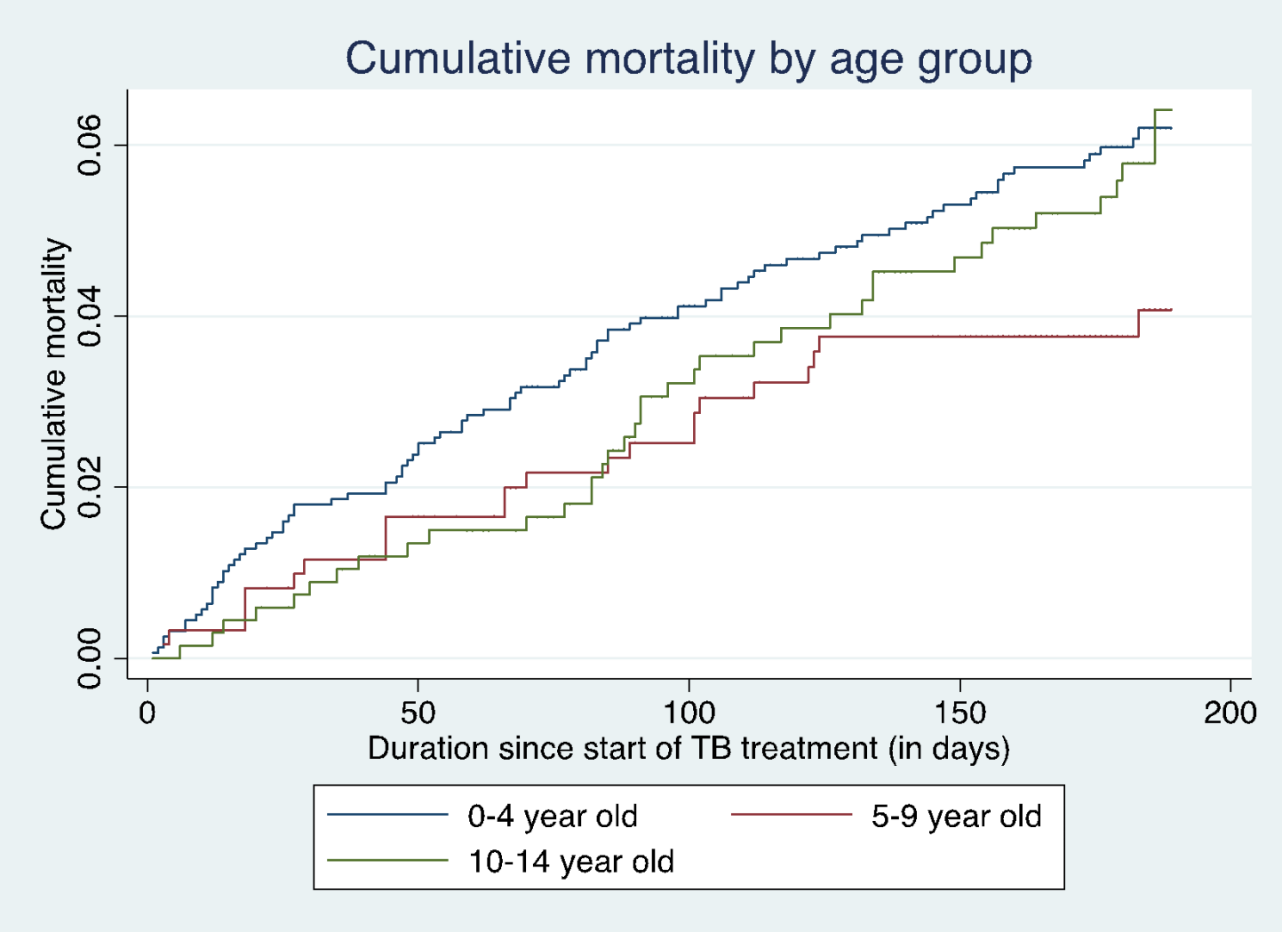
Cumulative hazard of death by age group (Log rank: Chi^2^ = 10.95, p = 0.004

Figure 1 shows the cumulative hazard of death by age group. The cumulative mortality was significantly higher in the 0–4 years age category than the 5–9 years and 10–14 years age categories (log rank test, chi^2^ = 10.95, p = 0.004). The cumulative mortality curves of the 5–9 and 10–14 age groups were overlapping, signifying no significant difference between the 2 groups.

**)**

## Discussion

This is the first nationally representative study of pediatric TB outcomes in Botswana. We report on 6004 patients from all public health facilities in the country seen over a 12-year period from 2008 to 2019. More than half of these notifications were under the age of 5 years. This is expected, as younger children are more likely to progress from infection to active disease due to their immature immune systems [14]. However, this is concerning as infection in this age group is almost always a marker of recent or ongoing transmission in the community and not reactivation of latent disease, necessitating the need to intensify community infection prevention and control measures [19]. Other African studies have reported a high proportion of paediatric cases in this age category [14, 20, 21]. The majority of cases in our study had pulmonary TB with extrapulmonary TB (EPTB) accounting for 21.7% of cases. This proportion is higher than what has been reported in Ghana and Uganda [14, 16]. The high proportion of EPTB in our study may be due to a higher HIV burden in Botswana [22].

In this study, the overall treatment success rate was 93.1%. The unfavorable outcomes of death, loss to follow-up and treatment failure occurred in 6.9% of cases. The mortality rate was relatively low at 3.4%. Similar to previous studies, the high success rates were mainly driven by treatment completion [13, 14, 16]. The cure rate was only 8.2%. The low cure rates may be due to difficulties with sputum collection, especially at the end of TB treatment in the paediatric population. The outcomes are significantly better than what was reported in Botswana in 2008. In the electronic record review of pediatric TB outcomes in Botswana from 1998 to 2002, the TB treatment success rate was 67% and the mortality rate was 10.5% [13]. Our results demonstrate a significant improvement from these early findings. This improvement is attributable, at least in part, to advances in pediatric TB diagnosis and child-friendly treatment and care. Several interventions have been introduced in the Botswana national TB program since the 2018 review. These include gene Xpert and direct observed therapy [22]. Advances in HIV diagnosis, treatment, and care may also have contributed to improved pediatric TB treatment outcomes. This is because HIV/AIDS is significantly associated with TB mortality [13, 18]. Furthermore, programmatic management of TB at the district level has been enhanced through bi-annual training of TB coordinators and clinicians with support from developmental partners.

Our findings showed better outcomes than what has been reported in other African countries [23, 24]. In Harare, Zimbabwe, surveillance data showed that 57% of patients had good treatment outcomes (cured or completed treatment), while pretreatment sputum positivity was a predictor of poor outcomes [25]. In Mozambique, the treatment success rate was 88% while the death rate was 4% [26]. Still, in Mozambique, the TB treatment success rate was 83.6% in a hospital-based cohort[27]. However, the outcomes in our study are significantly worse than what has been reported in European countries. The mortality rates were also very low in a cohort of 3563 pediatric TB patients in England and Wales. In this study of children under 16 years of age from 1999 to 2006, the mortality rate was < 1% [19]. This demonstrates that while there have been improvements of TB outcomes in our setting; we are still lagging behind high-resource countries, especially in minimizing pediatric TB mortality. The reason for this is likely multifactorial and may be influenced by multiple social determinants of health.

The mortality rates were highest in the 0–4 years age group. This is consistent with findings from Uganda and Ethiopia where children under 5 years had the worst treatment outcomes [16, 24]. Other studies have had similar findings [6, 14, 15]. TB in young children can be difficult to diagnose due to its paucibacillary nature. This may result in late diagnosis when complications have already set in. The difficulty in diagnosis may also lead to misdiagnosis of TB in this age group. This would result in inappropriate TB treatment and failure to manage alternative diagnoses. There is also evidence that TB in young children is more likely to present with severe disease [28, 29]. Our study adds to the evidence of disproportionate mortality in young children. In our setting of high HIV and TB prevalence, clinicians should have a low threshold to investigate TB in young children. Targeted public health interventions are also needed to address pediatric TB in general and under-5 TB in particular. This requires collaboration with child health and HIV programs.

In addition to the age 0–4 years category, the significant predictors of unfavorable outcomes in the total cohort were positive HIV status, unknown HIV status, and retreatment category. Positive HIV status is an established predictor of poor outcomes including mortality [15, 15– 33]. The higher mortality in HIV-positive children can be explained by immunosuppression, opportunistic infections, atypical presentation, drug-drug interactions and poor adherence [6, 14]. Our study underlines the need to optimize HIV prevention, treatment, and care. Positive HIV status has been identified as a significant predictor of mortality in other studies in Sub-Saharan Africa. In a retrospective study of paediatric TB outcomes in Accra, Ghana, the mortality was almost 4 times in HIV-positive cases than in negative cases [14]. In a Nigerian cohort, positive HIV status was associated with a 20% increase in the odds of mortality [15]. Other predictors of unfavorable paediatric TB outcomes have been identified elsewhere. In a study of TB outcomes in Ethiopia, patients with an unknown HIV status were about 2.5 times more likely to have unsuccessful treatment outcomes than HIV negative patients [24].

In a Pakistan study, fever and examination suggestive of abdominal TB were predictors of bad outcomes while coughing and being treated in a rural facility were associated with lower risk of a bad outcome [34]. Unfortunately, many of these factors could not be assessed in our study. The program data had limited variables and had other data quality issues including data completeness. Future prospective or facility-based studies should investigate the association between these factors and paediatric TB outcomes.

## Limitations

This study has some limitations. The use of routinely collected program data meant only a limited number of variables could be used in the descriptive and inferential analysis. Notably, data on the diagnosis of TB, e.g., clinical and radiological findings, for individual patients were not captured on the electronic medical records system. The only information on the diagnosis is about microscopy results. Furthermore, there is no data on whether the facilities were rural or urban, private or public, etc. Since there were no facility level identifiers, we could not control for clustering by facility in the multiple regression model. The routinely collected data has challenges of completeness and accuracy. This can cause misclassification bias. Misclassification could also result from relying on the TB coordinators’ determination of treatment outcomes. The use of program data also means underreporting cannot be ruled out. Despite these limitations, this is a vital study that offers a nationally representative investigation of paediatric TB outcomes and associated factors in Botswana.

## Conclusion

In our setting of high TB and HIV burden, TB outcomes were mostly favorable with relatively high treatment success rates. The outcomes were comparable to many similar settings. Almost half of the paediatric cases were children under the age of 5 years. This age group also had the highest mortality. The outcomes continue to lag behind those of high-income countries. The factors associated with the unfavorable outcomes were 0–4 years age group, positive and unknown HIV status and retreatment category. A large proportion of the records were of cases with unknown HIV status. Public health interventions should particularly target younger children, those with a positive or unknown HIV status, and those who are retreated. HIV testing should be optimized in this age group. Further studies are needed to understand the factors associated with TB treatment outcomes in children.

## Electronic supplementary material

Below is the link to the electronic supplementary material.

Supplementary Material 1

## Data Availability

All data generated or analyzed during this study are included in this published article (and it’s supplementary information files).
